# Multiple Sclerosis: Impact on Oral Hygiene, Dysphagia, and Quality of Life

**DOI:** 10.3390/ijerph17113979

**Published:** 2020-06-04

**Authors:** Francesco Covello, Giovanni Ruoppolo, Camilla Carissimo, Giulia Zumbo, Carla Ferrara, Antonella Polimeni, Iole Vozza

**Affiliations:** 1 Oral and Maxillo-Facial Sciences Department, Sapienza University of Rome, 00185 Rome, Italy; francesco.covello@uniroma1.it (F.C.); camilla.carissimo@gmail.com (C.C.); antonella.polimeni@uniroma1.it (A.P.); iole.vozza@uniroma1.it (I.V.); 2 Sensorial Organs Department, Sapienza University of Rome, 00185 Rome, Italy; giovanni.ruoppolo@uniroma1.it; 3 Public Health and Infectious Diseases Department, Section Health Statistics, Sapienza University of Rome, 00185 Rome, Italy; carla.ferrara@uniroma1.it

**Keywords:** multiple sclerosis, dysphagia, oral health, oral hygiene

## Abstract

Multiple sclerosis (MS) is an autoimmune disease in which the immune system reacts by damaging the central nervous system, specifically myelin and oligodendrocytes. It is the most debilitating neurological disease among young adults, causing personal, familiar, social, and professional limitations. Multiple sclerosis can cause disturbances in the orofacial district, due to a demyelination process on the nerves of the head and neck district. The aim of this study was to evaluate the oral health status, dysphagia, and quality of life of patients affected by MS. For this study, 101 patients aged between 12 and 70 (47 males, 54 females) affected by MS were selected, and three questionnaires were handed out and anonymously filled in by them: An oral hygiene test, DYMUS (DYsphagia in MUltiple Sclerosis), and IOHIP-14 (Italian version Oral Health Impact Profile). Through the analysis of the questionnaires it was possible to observe pathological conditions, such as gingival inflammation, xerostomia, dysphagia, neuralgia, and dysarthria. Through the analysis it was possible to outline how the roles of a medical team, composed of a dentist, otolaryngologist, and dental hygienist, are fundamental in coping with other medical figures, during the whole development of the diseases, as well as to prevent possible complications.

## 1. Introduction

Multiple sclerosis (MS) is an autoimmune disease in which the immune system reacts by damaging the central nervous system, specifically myelin and oligodendrocytes [1]. In fact, the immune system triggers a reaction that ends with the formation of sclerotic plaques that can be shown in different areas of the central nervous system (CNS) [2]. It is the most disabling neurological disease in young adults that causes considerable personal, familiar, social, and professional limitations [3]. It was often stated that multiple sclerosis etiology is unknown; nevertheless, Epstein–Barr virus (EBV), sun (ultraviolet B rays), smoking, and vitamin D, combined with the genetic characteristics of the individual, play an important role in the development of multiple sclerosis [4,5]. Children born in Europe from migrants coming from countries with a low risk of incidence of MS, such as occidental Indies, have high risks of developing it. Consequently, environmental factors have been demonstrated to be more important than genetics in the etiology of MS, supporting studies regarding prevention over environmental risk factors [2]. MS is more common in females and the ratio between the two sexes is close to 3:1 in most developed countries [4].

Disease progression in MS may manifest as numerous sensory and motor disturbances in the orofacial region; these manifestations of MS can occur following a demyelination of the nerves of the head and neck region [3,6]. The main clinical presentations in the head and neck region are cranial neuralgias, facial paralysis, temporomandibular disorders, visual complication, dysphagia, and dysarthria. Cranial neuralgias (CNs), including trigeminal neuralgia (TN), glossopharyngeal neuralgia (GN), and occipital neuralgia (ON), are typical expressions of neuropathic pain in MS and are characterized by paroxysmal painful attacks of electric shock-like sensations that may be spontaneous or evoked by innocuous stimuli in specific trigger zones. Demyelination is thought to play an important role in the origin of neuralgic pain in symptomatic cranial neuralgias in MS. When affecting the motor nerves, it may present in the orofacial region as muscle weakness, tremor, hemi facial spasms, and myokymia (involuntary facial muscle contractions) [7,8].

Furthermore, sensory neuropathy secondary to MS may present as bilateral, progressive, and often irreversible paresthesia commonly involving the second and third divisions of the trigeminal nerve. These may be accompanied by extra oral or intraoral numbness, tingling, or pain. There is a high prevalence of temporomandibular joint (TMJ) disorders’ symptomatology in MS (pain on mouth opening, difficulty in mouth opening, TMJ sounds), which may be attributed to the underlying myofascial and neck pain [9].

Painful tonic spasms of the facial muscles are specific to MS. These are unilateral or bilateral, stereotyped, and involuntary muscle contractions that last less than 2 min and may manifest several times a day. They can be triggered by touch, movement, hyperventilation, or emotions, and are, though seldom, preceded by a “somesthetic aura”. They may start from the face and spread to the adjacent part of the body. The spasms originate in the central nervous system from hyperactivity in the central motor fibers, caused by lesions in the internal capsule, cerebral peduncle, medulla, or spinal cord.

Several studies reported the impact of orofacial symptoms on the quality of life of patients affected by MS [7]. The above-described symptoms, plus spasticity, ataxia, tremor, fatigue, depression, and progressive disability, have an impact on the individual’s ability to maintain oral health, cope with dental treatment, and access dental services. Patients with numbness or paresthesia in the arms and hands may have difficulty holding items, such as a toothbrush. The tremor can affect the head, jaw, lips, and speech, and cause a loss of coordination. Depression decreases motivation for self-care [10].

Manual dexterity is decreased in the patient severely affected by MS, and loss of muscular coordination results in increased difficulty maintaining adequate oral hygiene [11].

Considering that the lack of muscular coordination connected to tremors shown in patients affected by MS can influence oral hygiene, orofacial aspects connected to this aspect have to be considered by dentists [12]. 

Although the oral effects of MS are significant, in the international literature, there are only a few studies considering this aspect. The aim of this study was to evaluate the oral health status, dysphagia, and quality of life of patients affected by MS, and to outline how oral care in MS patients can play a fundamental role in the management of the disease-associated disturbances and in contributing to enhancing the general wellbeing of the patient.

## 2. Materials and Methods 

In this study, 101 patients aged between 12 and 70 (47 males, 54 females) affected by MS were selected. Patients were selected between 14 November 2018 and 24 July 2019, in the “Multiple Sclerosis” clinic of the Department of Neurology and Nervous System Diseases of “Policlinico Umberto I” of Rome.

The protocol of our trial is consistent with Good Clinical Practice standards of the European Union set out in the Declaration of Helsinki of 1975. The study was approved by the Institutional Review Board of territorial NHS facilities (130/13). 

Three questionnaires to be filled in were handed out to the patients selected: An oral hygiene test, DYMUS (DYsphagia in MUltiple Sclerosis), and IOHIP-14. The samples were taken anonymously. 

The three questionnaires correspond to three models that already exist. The questionnaire regarding the general status of the oral cavity and the oral hygiene habits and its quality is a form made up by 15 questions that aimed to evaluate several aspects: The frequency of the oral hygiene daily care, the self-evaluation of the manual skills in performing it, the type of tools used for it, the presence of symptoms related to periodontal disease (gingival bleeding, tooth mobility), the presence of xerostomia, the presence of dentinal sensitivity, the frequency of dental check-ups or professional oral hygiene sessions, and the hygiene condition of prosthetic devices when present.

The second questionnaire, the DYMUS, allows screening for the evaluation of the presence of dysphagia [13]. It is formulated as 10 questions that only allow positive or negative answers related to the health status of the patient. Every question is aimed at analyzing the characteristics of the dysphagia related to the ingestion of solid or liquid substances. In the case of positive outcome, the patient needs to be referred to a podiatrist.

Lastly, IOHIP-14 (14 items Oral Health Impact Profile—Italian version) assesses how oral health influences the quality of life of the patients. In OHIP-14, which is a synthesis of OHIP-49, 14 questions were extracted from the initial 49, in order to get a faster and more intuitive preformed form [14]. Through this model, the patient can self-evaluate the perception of their oral health status by assigning values from 0 to 4 (0 = never; 1 = almost never; 2 = sometimes; 3 = quite often; 4 = very often). The goal of the analysis of this model is to analyze 7 fundamental aspects: Functional limitations (chewing difficulties, tasting worsened ability), physical pain (dentinal sensitivity, muscular tension sensation), psychological discomfort (self-awareness), physical disability (change of diet), psychological disability (reduced focusing ability), social disability (avoiding social interaction, causing interruption during meals), and handicap. Therefore, IOHIP-14 is a subjective indicator.

Descriptive statistics were computed for each item and the percentage of participants’ answers to each item was calculated. The analysis of the data was performed using SPSS 14.0 for Windows (SPSS Inc., Chicago, IL, USA). The data regarding the difference between men and women were then statistically analyzed using Fisher’s exact test.

## 3. Results

In total, 101 patients affected by MS were selected for this study, and were aged between 12 and 70, with 47 males and 54 females. Three questionnaires were handed out and anonymously filled by the patients: An oral hygiene test, DYMUS (DYsphagia in MUltiple Sclerosis), and IOHIP-14 (Italian version Oral Health Impact Profile). 

Through the analysis of the data collected in our study, we were able to make the following observations. 

### 3.1. Oral Hygiene Habits and Its Quality Questionnaire

If at first, we consider the data regarding the questionnaire on oral hygiene habits and its quality, together with the general status of the oral cavity (Table 1), it can be outlined how 58% of the patients practice oral hygiene procedures twice a day, while none of the subjects analyzed declared not performing it. Another remarkable finding of this trial is that 50% of the patients evaluated their manual skills in domestic oral hygiene as good, while 26% evaluated them as sufficient. Besides, 71% of them declared using a manual toothbrush, while the remaining 13% the electric one. Concerning the use of dental floss, our data showed that 12% of them often use it, while 52% of them almost never use it. Pipe cleaners are used daily by just 8% of the selected patients, while 70% never use them. The tongue is brushed daily by 8% of the interviewed patients, while 51% affirmed that they brush their tongue almost never. Our analysis showed that 26% of the patients wear a denture and that 19% of them clean it daily. Furthermore, 40% of the subjects referred halitosis. Concerning the use of mouthwash, 34% of the patients observed never or almost never use it, 36% use it sometimes, while 7% use it daily. In total, 2% of patients reported daily gum bleeding, 9% of which is often, and 46.5% is sometimes. Dental mobility of different grades was observed in 8% of the patients. Dentinal sensitivity to thermal and chemical stimuli was reported by 40% of the patients, with 18% of them defining it as strong, while 34% of the patients declared they did not suffer from dentinal sensitivity. Xerostomia was observed in 62% of the examined patients, being absent in the remaining 38%. 

In total, 65% of the examined subjects never feel numb or paresthesia on the lips and/or gums, while 30% sometimes feel this sensation. Just 6% of the patients regularly (every 3/6 months) undergo dental treatments, 24% do check-ups every 6/12 months, 43% do them less often, that is to say every 12/18 months, and 27% do not have check-ups for over 18 months.

In the comparison between males and females, the results obtained from this questionnaire were statistically analyzed using Fisher’s exact test and the differences between males and females were found to be non-significant.

### 3.2. DYMUS Questionnaire

We will now take into consideration the results of the last questionnaire, DYMUS, whose full outcomes are reported in Table 2. It was outlined that 25% of the interviewed subjects have this problem with liquid substances. The percentage of patients affirming to have the sensation of “globus pharyngis” is equal to 26%.

The percentage of patients that declared coughing or having an asphyxiation sensation when eating solid or liquid food is equal to 24%. 

In total, 22% of the patients need to swallow several times before being able to completely ingest solid food, 21% need to cut food in smaller pieces in order to facilitate the swallowing, and 26% need to sip several small times when drinking.

The percentage of patients reporting weight loss in the last six months is equal to 18%, although some of them affirm having done that on purpose with the help of a specific diet prescribed by their doctor.

Additionally, the results from the DYMUS questionnaire were statistically analyzed using Fisher’s exact test and the differences between males and females were found to be non-significant.

### 3.3. IOHIP-14—Italian Oral Health Impact Profile

The analysis of the data of IOHIP-14 will follow (Table 3).

Difficulty in pronouncing words was sometimes experienced by 26.8% of the patients. Worsening of taste perception was noticed, with more or less frequency, in 12% of the patients. In total, 16% of the patients complained about pain in the mouth, while 77% of them did not report this.

In total, 14% of patients feel discomfort in eating, while 22% report a sensation of muscular tension, 5% frequently, and 10% very often. In total, 14% of the patients consider their diet as sometimes unsatisfying, while 12% of them consider it as unsatisfying rarely. In total, 8% of the patients declared they sometimes need pauses during meals due to dysphagia. In the self-analysis regarding the difficulty in relaxing, after craniofacial disturbances showed up, including the muscular ones, 21% of the patients declared experiencing difficulties with this sometimes, while 12% of them more often. When measuring the irritability due to the conditions of the oral cavity, 21% of the subjects sometimes feel this disturbance; 17% of them experienced difficulties in working due to these disturbances, that is why 23% of the patients consider their quality of life as less satisfying; 2% of them believe it to be totally uncapable of practicing any activity.

All of the above-mentioned results from IOHIP-14 were statistically analyzed using Fisher’s exact test: The differences between males and females were found to be significant (*p* = 0.0349) in the difficulty in relaxing but not for the other parameters taken into consideration.

## 4. Discussion

In patients affected by MS, a gradual and progressive limitation of functional skills is observed, consequently leading to disability. Clinical symptoms of MS include motor deficits, vision deficits, bulbar symptoms, cognitive dysfunctions, and mental disturbances, which might interfere with oral hygiene. Furthermore, the progressively increasing functional disability, in association with the drugs used for MS, can cause a greater incidence of pathologies in the mouth and teeth [15].

For this reason, is important to consider the oral health status, with this certainly being connected to the quality of life.

From the results of the questionnaire regarding domestic oral hygiene, it was found that 58% of the patients brush twice a day, 71% of them use a manual brush, and nearly 13% use an electric one.

An electric brush can reduce the quantity of plaque from 7% to 58%, gum inflammation from 17% to 20%, and gum bleeding to 85%, and it is also more efficient than a manual brush [16].

Concerning the use of dental floss, 12% of the subjects analyzed use it daily. Pipe cleaners are used daily by just 8% of the patients selected. The lack of awareness of the need to use dental aids, or incorrect use of them, might create a series of oral complications, with systemic consequences. 

Therefore, explaining to the patient the proper procedures in utilizing the abovementioned devices is fundamental, in order to prevent gum and periodontal inflammation [12,17,18], but it is also important that these conditions are treated properly, with scaling and root planning (SRP), eventually combining them with probiotics [19]. In fact, according to recent studies, probiotics might create a synergic effect with the current therapies used to cure MS [20].

Due to inefficient plaque removal, 9% of the selected subjects of this trial declared that they often notice gum bleeding. In the literature, there are studies that report a gum bleeding incidence of between 5% and 15% in patients affected by MS [21].

Inefficient oral hygiene operation could be caused by the poor manual skills related to the disease. 

In fact, among the patients selected in our trial, 26% declared they had sufficient manual skills, while 4% declared them to be insufficient. In total, 40% of the subjects that filled in the questionnaire had halitosis. When analyzing these data, it would be appropriate to do a differential diagnosis among physiological halitosis, pseudo halitosis, and halitophobia, using different methods (organoleptic evaluation, gas chromatography, salivary ß-galactosidases activity) [22,23,24]. 

The study of Danesh-Sani [7] outlines the oral and craniofacial manifestation of MS. Among them, for instance, dysarthria was reported to be present in 42.1% of the cases. This result is not far from our analysis, where 38% of patients complained about this difficulty. Concerning dysphagia, the results of the study of Danesh-Sani [7] reported a percentage of patients that have this symptom equal to 26.6%, while in the study of Calcagno, it is equal to 34.3% [25]. In our study, dysphagia was observed in 15% of the subjects analyzed. According to a study of Hollaar V. et al. of 2015 [26], dysphagia can cause a deficit in the social life of the patient, bringing an infection of the airways. The prevention of complications is the primary goal in patients with dysphagia, in order to avoid aspiration pneumonia, malnutrition, and dehydration and then an intellectual and body development deficit in children, or emotional impairment and social restriction [27].

Due to dysphagia, 15% of the patients filling in our questionnaire affirmed they needed to pause when eating. Regarding this, our second questionnaire, DYMUS, highlighted that 25% of the interviewed patients struggle in swallowing solid food, while 21% have this problem with liquids. 

All these problems inevitably affect not only the physical aspect but also the psychological one. 

Another relevant aspect of our research is related to psychological-related problems, which might be caused by MS orofacial disturbances. Among them, we can mention the worsening of taste perception, observed in 17% of our sample; discomfort in eating, declared by 18% of the subjects; and pain in the oral cavity (neuralgia), shown by 28% of the patients. Regarding neuralgia, it is important to remark on how important the role of the dentist in the early diagnosis of MS is by identifying the characteristics of the pain caused by trigeminal neuralgia. In fact, the latter is typically described as short-lasting heavy paroxysmal pain that is caused either by the pression or by chewing forces. Besides, it is experienced several times a day and it is usually bilateral [7].

Considering the above-discussed aspects and the broad spectrum of symptoms, conditions, and impairment that MS can cause, it is clear that the management of this condition requires a multidisciplinary team approach of health care providers and allied health professionals. The dentist, otolaryngologist, and dental hygienist have a fundamental role in intercepting or properly treating MS-related disturbances in the oral cavity, through a specific program of professional and domestic therapies, coordinating with the physician in order to constantly monitor the progression of MS and adapt the therapies to it. 

The entire oral health care team at the outpatient setting must participate in all features of oral care, such as advice, skills, motivation, verbal reassurance, support throughout the treatment phase of the patient, as well as after, to contribute to good oral health, improve patient attendance at oral health clinics, and enhance the general wellbeing of the patient [6,10,28]. 

## 5. Conclusions

From the results obtained in this trial, we can state that in patients affected by MS, we noticed the presence of disturbances, such as dysphagia, neuralgia, dysarthria, gum inflammation, and xerostomia, that negatively interfere with the quality of life, although not more than the disease itself already does. Therefore, it is possible to highlight how important the role of a medical team, composed of a dentist, otolaryngologist, and dental hygienist, is in intercepting or properly treating MS-related disturbances in the oral cavity, through a specific program of professional and domestic therapies. It would be profitable if more studies can pursue and analyze craniofacial disturbs shown during the development of MS, in order to obtain statistically significant data.

## Figures and Tables

**Table 1:** Oral Hygiene Habits and Its Quality Questionnaire Results

Oral Hygiene Questionnaire				
Total *N*. of Patients (*N*. of Males; *N*. of Females)				
	**Three or More**	**Two**	**One**	**None**
How much time do you dedicate daily to your oral hygiene?	32 (17;15)	59 (25;34)	10 (5; 5)	0 (0; 0)
	**Excellent**	**Good**	**Sufficient**	**Insufficient**
Manual skills in performing domestic oral hygiene	19 (7; 12)	51 (24; 27)	27 (13; 14)	4 (3; 1)
	**Both**	**Electrical**	**Manual**	**I do not have toothbrush**
Do you use electrical and/or manual toothbrush?	16 (6; 10)	13 (9; 4)	72 (32; 40)	0 (0; 0)
	**Daily**	**Frequently**	**Sometimes**	**Nearly or never**
Do you use dental floss?	12 (7; 5)	12 (6; 6)	24 (9; 15)	53 (25; 28)
Do you use pipe cleaners?	6 (3; 3)	9 (3; 6)	15 (8; 7)	71 (35; 38)
Do you clear your tongue?	9 (5; 4)	9 (5; 4)	31 (9; 22)	52 (28; 24)
	**Always**	**Often**	**Sometimes**	**Nearly or never**
Do you bleed during when brushing of your teeth?	2 (0; 2)	9 (7; 2)	47 (20; 27)	43 (20; 23)
Do you feel sometimes your mouth dry?	4 (3; 1)	13 (7, 6)	46 (25; 21)	38 (12; 26)
Do you suffer from halitosis?	0 (0; 0)	12 (7; 5)	41 (20; 21)	48 (20; 28)
Do you feel your lip, gum, tongue, mucosa or other mouth areas are numb?	0 (0; 0)	7 (5; 2)	31 (20; 10)	63 (21; 42)
	**Yes, a lot**	**Enough**	**Not a lot**	**No**
Do you feel mobility in your teeth?	3 (2; 1)	8 (5; 3)	15 (9; 6)	75 (31; 44)
Do you feel dental sensitivity with hot, cold and sweet?	8 (3; 5)	18 (10; 8)	41 (18; 23)	34 (16; 18)
	**Every 3\6 months**	**Every 6\12 months**	**Every 12\18 month**	**Beyond 18 months**
How often you undergo professional oral hygiene’s appointment or a dental examination?	6 (4; 2)	24 (10; 14)	43 (17; 26)	28 (16; 12)
	**Daily**	**Often**	**Sometimes**	**Nearly or never**
Do you use mouthwash?	7 (2; 5)	22 (8; 14)	37 (17; 20)	35 (20; 15)
	**Often**	**Sometimes**	**Nearly or never**	**I do not have a denture**
Do you clean your denture?	20 (9; 11)	0 (0; 0)	7 (2; 5)	74 (36; 38)

**Table 2:** DYMUS Questionnaire Results (DYsphagia in MUltiple Sclerosis)

DYMUS Questionnaire						
Characteristics of the Interviewed Subjects	Males		Females		Total	
	NO	YES	NO	YES	NO	YES
Difficulty in swallowing solid food	37	10	39	15	76	25
Difficulty in swallowing liquids	36	11	44	10	80	21
Sensation of “globus pharyngis” when swallowing	35	12	40	14	75	26
Sensation of food stuck in the throat when swallowing	37	10	39	15	76	25
Coughing or asphyxia sensation when swallowing solid foods	34	13	43	11	77	24
Coughing or asphyxia sensation when swallowing liquid foods	36	11	41	13	77	24
Need of swallowing several times before ingesting solid food	37	10	42	12	79	22
Need of cutting food in several smaller pieces before swallowing	36	11	44	10	80	21
Need of sipping several times when drinking	33	14	42	12	75	26
Weight loss	36	11	37	17	73	28

**Table 3:** IOHIP-14—Italian Oral Health Impact Profile Results

IOHIP-14 (Italian Oral Health Impact Profile)					
Total *N*. of Patients (*N*. of Males; *N*. of Females)					
	Never (0)	Rarely (1)	Sometimes (2)	Frequently (3)	Very Often (4)
Difficulty in pronunciation	35 (16; 19)	27 (11; 16)	27 (14; 13)	10 (5; 5)	2 (1; 1)
Worsening of taste perception	66 (25; 41)	18 (12; 6)	12 (7; 5)	4 (3; 1)	1 (0; 1)
Pain	58 (26; 32)	20 (11; 9)	16 (7; 9)	5 (2; 3)	2 (1; 1)
Discomfort in eating	63 (26; 37)	20 (12; 8)	14 (8; 6)	3 (1; 2)	1 (0; 1)
Awareness	52 (20; 32)	13 (5; 8)	20 (12; 8)	9 (7; 2)	7 (3; 4)
Sensation of tension	52 (22; 30)	12 (6; 6)	22 (12; 10)	5 (1; 4)	10 (6; 4)
Unsatisfactory diet	67 (29; 38)	12 (8; 4)	14 (9; 5)	4 (0; 4)	4 (1; 3)
Meal interruption	76 (32; 44)	13 (10; 3)	8 (4; 4)	2 (0; 2)	2 (1; 1)
Difficulty in relaxing	40 (15; 25)	18 (10; 8)	21 (14; 7)	12 (2; 10)	10 (6; 4)
Embarrassment	60 (26; 34)	15 (10; 5)	19 (7; 12)	5 (3; 2)	2 (1; 1)
Irritability	44 (17; 27)	15 (11; 4)	21 (9; 12)	13 (6; 7)	8 (4; 4)
Difficulty in working	50 (23; 27)	20 (10; 10)	17 (8; 9)	9 (5; 4)	5 (1; 4)
Less satisfying life	40 (17; 23)	24 (13; 11)	23 (12; 11)	8 (2; 6)	6 (3; 3)
Totally uncapable of performing activity	73 (34; 39)	12 (5; 7)	11 (6; 5)	3 (2; 1)	2 (0; 2)
